# Supplementary material for: The aroma of TEMED as an activation and stabilizing signal for the antibacterial enzyme HEWL
Source: PLoS One. 2020 May 19;15(5):e0232953. doi: 10.1371/journal.pone.0232953 (PMC7236982; doi:10.1371/journal.pone.0232953)
Supplement: S1 Fig — (a,e) HEWL at pH 8.6 (PDB ID 6ABN), (b,f) TEMED-co (PDB ID 6ADF), (c,g) TEMED5h (PDB ID 6AEA) and (d,h) TEMED24h (PDB ID 6AD5). Structure graphics were generated using Coot from CCP4 package version 2.10.7 [17]. (DOCX) [file pone.0232953.s001.docx]

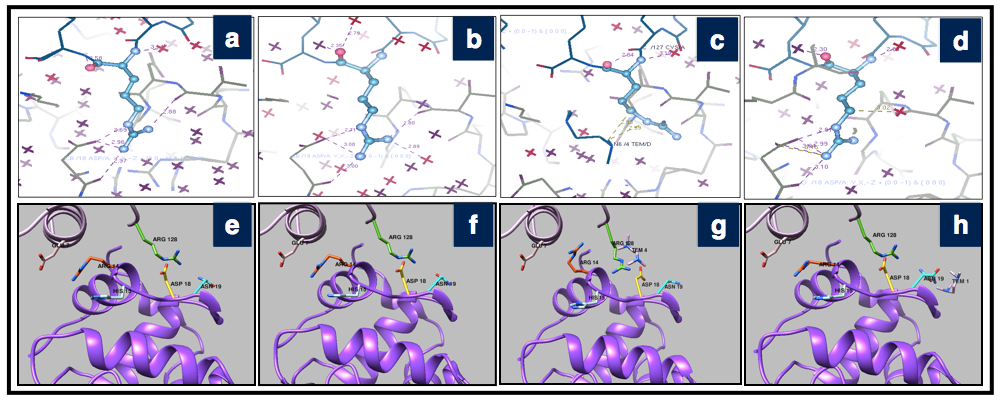


**Figure S1. Shifts or rotamers of Arg128 and Asn19 in the different structures.** **(a,e)** HEWL at pH 8.6 (PDB ID 6ABN), **(b,f)** TEMED-co (PDB ID 6ADF), **(c,g)** TEMED5h (PDB ID 6AEA) and **(d,h)** TEMED24h (PDB ID 6AD5). Structure graphics were generated using Coot from CCP4 package version 2.10.7.
