# Supplementary material for: The aroma of TEMED as an activation and stabilizing signal for the antibacterial enzyme HEWL
Source: PLoS One. 2020 May 19;15(5):e0232953. doi: 10.1371/journal.pone.0232953 (PMC7236982; doi:10.1371/journal.pone.0232953)

**Figure S2. Electron density maps showing binding of TEMED to HEWL. (a-d)** Binding of four TEMED molecules to HEWL after 5h incubation. (**e and f)** Binding of two TEMED molecules to HEWL after 24h incubation. (**g)** Binding site of a single TEMED in HEWL upon co-crystallisation. The σA-weighted 2Fobs−Fcalc maps were contoured at 0.5 sigma and generated in Coot from CCP4 package version 2.10.7.


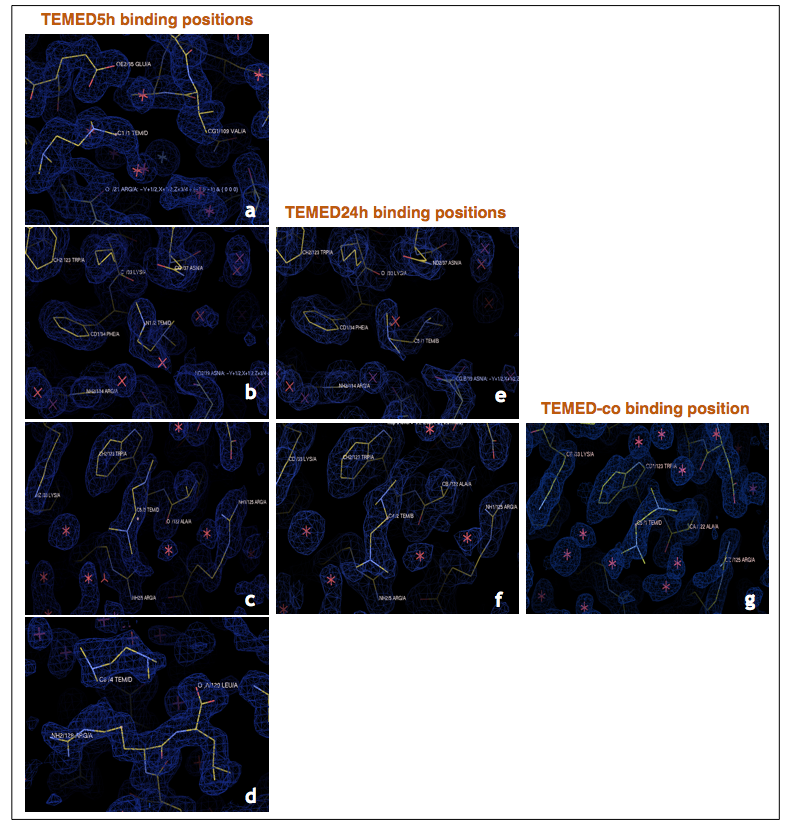

Supplement: S2 Fig — (a-d) Binding of four TEMED molecules to HEWL after 5h incubation. (e and f) Binding of two TEMED molecules to HEWL after 24h incubation. (g) Binding site of a single TEMED in HEWL upon co-crystallisation. The σA-weighted 2Fobs−Fcalc maps were contoured at 0.5 sigma and generated in Coot from CCP4 package version 2.10.7 [17]. (DOCX) [file pone.0232953.s002.docx]
