# Supplementary material for: The aroma of TEMED as an activation and stabilizing signal for the antibacterial enzyme HEWL
Source: PLoS One. 2020 May 19;15(5):e0232953. doi: 10.1371/journal.pone.0232953 (PMC7236982; doi:10.1371/journal.pone.0232953)
Supplement: S4 Fig — The σA-weighted 2Fobs−Fcalc maps (in blue mesh) were contoured at 0.5 and 1.0 sigma levels and the σA-weighted Fobs-Fcalc maps (in green and red mesh) were contoured at a default sigma level of 3.0. The maps were generated in Coot from CCP4 package version 2.10.7 [17]. (DOCX) [file pone.0232953.s004.docx]

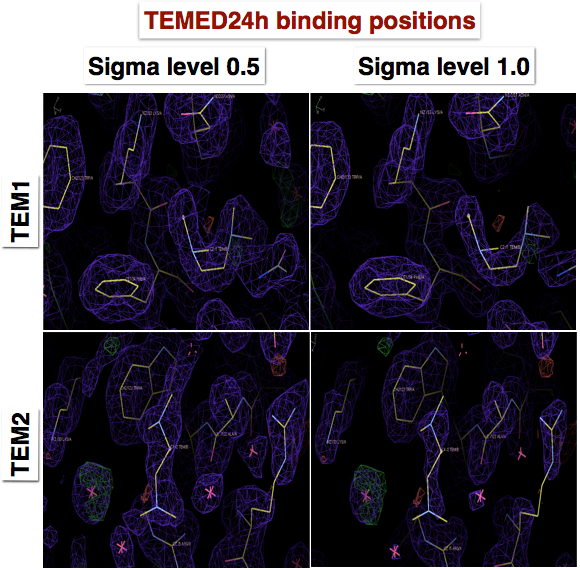


**Figure S4. Electron density map showing binding of TEMED to HEWL after 24h incubation.** The σA-weighted 2Fobs−Fcalc maps (in blue mesh) were contoured at 0.5 and 1.0 sigma levels and the σA-weighted Fobs-Fcalc maps (in green and red mesh) were contoured at a default sigma level of 3.0. The maps were generated in Coot from CCP4 package version 2.10.7.
