# Supplementary material for: The aroma of TEMED as an activation and stabilizing signal for the antibacterial enzyme HEWL
Source: PLoS One. 2020 May 19;15(5):e0232953. doi: 10.1371/journal.pone.0232953 (PMC7236982; doi:10.1371/journal.pone.0232953)
Supplement: S6 Fig — The wells contain the following: (Marker) Protein marker, (lane 1) Not-heated HEWL, (lane 2) Not-treated5h, (lane 3) Not-treated24h, and (lanes 8 and 9) TEMED5h and TEMED24h, respectively. Lanes 4–7 contain samples not relevant to this research article. This Supplementary Figure is provided since the original uncropped and unadjusted images underlying all blot or gel results are to be reported. (DOCX) [file pone.0232953.s006.docx]

**Figure S6. The inhibitory effect of aroma from TEMED on HEWL fibrillation as assessed by SDS-PAGE.** The wells contain the following: (Marker) Protein marker, (lane 1) Not-heated HEWL, (lane 2) Not-treated5h, (lane 3) Not-treated24h, and (lanes 8 and 9) TEMED5h and TEMED24h, respectively. Lanes 4-7 contain samples not relevant to this research article. *This Supplementary Figure is provided since the original uncropped and unadjusted images underlying all blot or gel results are to be* **
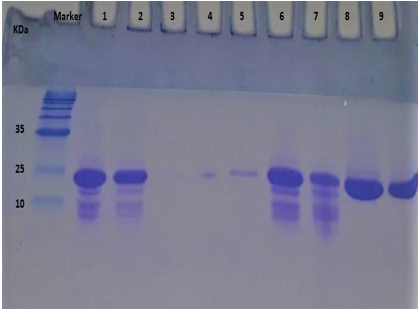
***reported.*
