# Supplementary material for: The aroma of TEMED as an activation and stabilizing signal for the antibacterial enzyme HEWL
Source: PLoS One. 2020 May 19;15(5):e0232953. doi: 10.1371/journal.pone.0232953 (PMC7236982; doi:10.1371/journal.pone.0232953)
Supplement: S1 Table — (DOCX) [file pone.0232953.s007.docx]

**Table S1.** **Various concentrations of TEMED used in this study using a vapour diffusion method.**

| **Reduced amount from 50 µl (initial volume)** | **Dilution factor** | **Final concentration of TEMED**  **(initial concentration divided by dilution factor)** |
| --- | --- | --- |
| **3 µl** | 3µl/4000µl= 1333times | 6.68M/1333=5.011mM |
| **10 µl** | 10µl/4000µl= 400times | 6.68M/400=16.07mM |
| **20 µl** | 20µl/4000µl= 200times | 6.68M/200=33.40mM |
| **30 µl** | 30µl/4000µl= 133times | 6.68M/133=50.11mM |
| **50 µl** | 50µl/4000µl= 80times | 6.68M/80=83.50mM |
