# Supplementary material for: The aroma of TEMED as an activation and stabilizing signal for the antibacterial enzyme HEWL
Source: PLoS One. 2020 May 19;15(5):e0232953. doi: 10.1371/journal.pone.0232953 (PMC7236982; doi:10.1371/journal.pone.0232953)
Supplement: S2 Table — (DOCX) [file pone.0232953.s008.docx]

**Table S2. Different rotamers of residues in HEWL in complex with TEMED and at pH 8.6.**

| **Samples** | pH 8.6 | TEMED-co | TEMED5h | TEMED24h |
| --- | --- | --- | --- | --- |
| **PDB IDs**  Rotamers | 6ABN | 6ADF | 6AEA | 6AD5 |
| Asn19 | **NO-Rotamer** | **NO-Rotamer** | **NO-Rotamer** | **Rotamer** |
| Asn44 | **NO-Rotamer** | **NO-Rotamer** | **Rotamer** | **Rotamer** |
| Ile55 | **Rotamer** | **Rotamer** | **NO-Rotamer** | **NO-Rotamer** |
| Ser85 | **Rotamer** | **Rotamer** | **Rotamer** | **NO-Rotamer** |
| Lys97 | **Rotamer** | **Rotamer** | **NO-Rotamer** | **NO-Rotamer** |
| Val109 | **Rotamer** | **NO-Rotamer** | **NO-Rotamer** | **NO-Rotamer** |
