# Supplementary material for: The aroma of TEMED as an activation and stabilizing signal for the antibacterial enzyme HEWL
Source: PLoS One. 2020 May 19;15(5):e0232953. doi: 10.1371/journal.pone.0232953 (PMC7236982; doi:10.1371/journal.pone.0232953)
Supplement: S3 Table — (DOCX) [file pone.0232953.s009.docx]

**Table S3. B-Factor values for Arg14, Glu7 and His15, Asp18, Asn19 and Arg128 in the structures of HEWL in complex with TEMED and at pH 8.6.**

| **Samples** | pH8.6 | TEMED-co | **TEMED5h** | TEMED24h |
| --- | --- | --- | --- | --- |
| PDB ID | **6ABN** | **6ADF** | **6AEA** | **6AD5** |
| B-factor (Å^2^) Arg14 | 13.99 | 19.47 | 25.92 | 26.97 |
| B-factor (Å^2^) Glu7 | 9.59 | 12.32 | 15.91 | 15.26 |
| B-factor (Å^2^) His15 | 11.11 | 15.11 | 22.96 | 19.68 |
| B-factor (Å^2^) Asp18 | 11.75 | 12.84 | 21.24 | 22.60 |
| B-factor (Å^2^) Asn19 | 15.83 | 18.32 | 24.31 | 18.26 |
| B-factor (Å^2^) Arg128 | 14.82 | 14.89 | 30.63 | 38.73 |
