# Supplementary figures and images for: The aroma of TEMED as an activation and stabilizing signal for the antibacterial enzyme HEWL
Source: PLoS One. 2020 May 19;15(5):e0232953. doi: 10.1371/journal.pone.0232953 (PMC7236982; doi:10.1371/journal.pone.0232953)

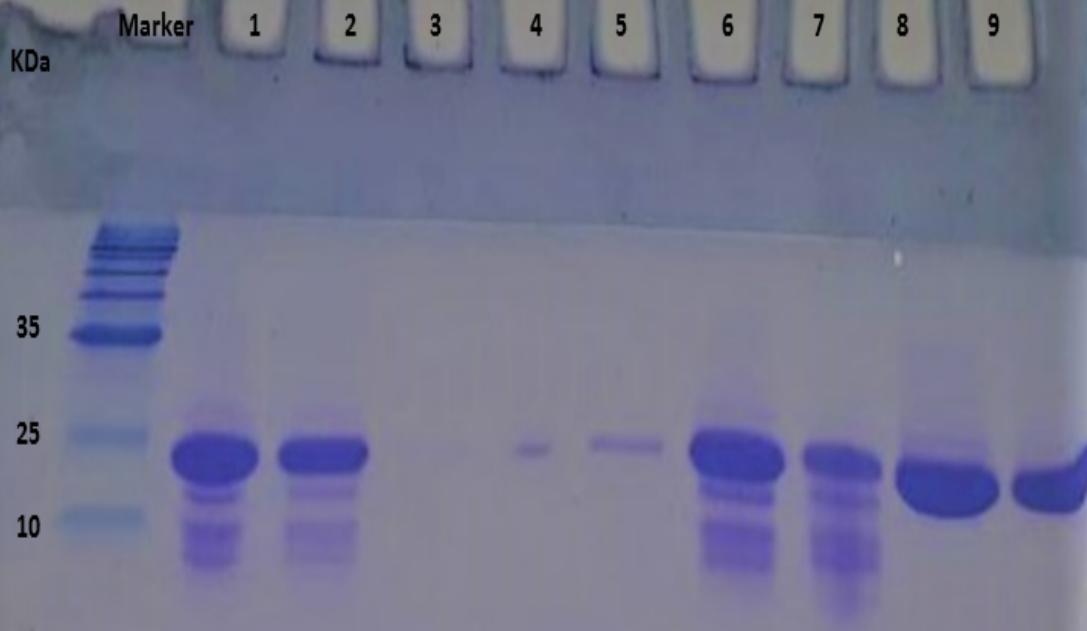

Supplement: S1 Data — (ZIP) [file pone.0232953.s010.zip › Raw_data_related_to_TEMED_paper_PLOS_ONE/gel.pdf]
