# Supplementary material for: The aroma of TEMED as an activation and stabilizing signal for the antibacterial enzyme HEWL
Source: PLoS One. 2020 May 19;15(5):e0232953. doi: 10.1371/journal.pone.0232953 (PMC7236982; doi:10.1371/journal.pone.0232953)
Supplement: Attachment — Submitted filename: Response to Reviewers_AS_ZS 13.docx [file pone.0232953.s011.docx]

Please include the following items when submitting your revised manuscript:

- A rebuttal letter that responds to each point raised by the academic editor and reviewer(s). This letter should be uploaded as separate file and labeled 'Response to Reviewers'.
- A marked-up copy of your manuscript that highlights changes made to the original version. This file should be uploaded as separate file and labeled 'Revised Manuscript with Track Changes'.
- An unmarked version of your revised paper without tracked changes. This file should be uploaded as separate file and labeled 'Manuscript'.

When submitting your revision, we need you to address these additional requirements.

- 1. Please ensure that your manuscript meets PLOS ONE's style requirements, including those for file naming. The PLOS ONE style templates can be found at

http://www.journals.plos.org/plosone/s/file?id=wjVg/PLOSOne_formatting_sample_main_body.pdf and http://www.journals.plos.org/plosone/s/file?id=ba62/PLOSOne_formatting_sample_title_authors_affiliations.pdf

Answer: Changes were applied to the main body of the manuscript.

- 2. Thank you for stating the following in the Acknowledgments Section of your manuscript:

"We acknowledge financial support of the Presidential Deputy for Science and Technology, the Institute for Research in Fundamental Sciences and the Iranian Light Source Facility for the opportunity to be users at the ALBA synchrotron program 2017 (proposal number ID2017062245). We acknowledge financial support of the OPEN SESAME for the two months student training fellowship for Miss Zahra Seraj (2018) and the Ministry of Science and Technology of Iran for six months research placement of Miss Zahra Seraj at University of Groningen, Netherlands."

We note that you have provided funding information that is not currently declared in your Funding Statement. However, funding information should not appear in the Acknowledgments section or other areas of your manuscript. We will only publish funding information present in the Funding Statement section of the online submission form.

Please remove any funding-related text from the manuscript and let us know how you would like to update your Funding Statement. Currently, your Funding Statement reads as follows:

"The author(s) received no specific funding for this work."

Answer: We have now moved the financial support statement to the funding section of the manuscript.

- 3. We note that Figure 8 in your submission contain copyrighted images. All PLOS content is published under the Creative Commons Attribution License (CC BY 4.0), which means that the manuscript, images, and Supporting Information files will be freely available online, and any third party is permitted to access, download, copy, distribute, and use these materials in any way, even commercially, with proper attribution. For more information, see our copyright guidelines: http://journals.plos.org/plosone/s/licenses-and-copyright.

We require you to either (1) present written permission from the copyright holder to publish these figures specifically under the CC BY 4.0 license, or (2) remove the figures from your submission:

a)	You may seek permission from the original copyright holder of Figure(s) [#] to publish the content specifically under the CC BY 4.0 license.

We recommend that you contact the original copyright holder with the Content Permission Form (http://journals.plos.org/plosone/s/file?id=7c09/content-permission-form.pdf) and the following text:

“I request permission for the open-access journal PLOS ONE to publish XXX under the Creative Commons Attribution License (CCAL) CC BY 4.0 (http://creativecommons.org/licenses/by/4.0/). Please be aware that this license allows unrestricted use and distribution, even commercially, by third parties. Please reply and provide explicit written permission to publish XXX under a CC BY license and complete the attached form.”

Please upload the completed Content Permission Form or other proof of granted permissions as an "Other" file with your submission.

In the figure caption of the copyrighted figure, please include the following text: “Reprinted from [ref] under a CC BY license, with permission from [name of publisher], original copyright [original copyright year].”

b)	If you are unable to obtain permission from the original copyright holder to publish these figures under the CC BY 4.0 license or if the copyright holder’s requirements are incompatible with the CC BY 4.0 license, please either i) remove the figure or ii) supply a replacement figure that complies with the CC BY 4.0 license. Please check copyright information on all replacement figures and update the figure caption with source information. If applicable, please specify in the figure caption text when a figure is similar but not identical to the original image and is therefore for illustrative purposes only.

Answer: The preference was to remove this Figure.

- 4. Please include captions for your Supporting Information files at the end of your manuscript, and update any in-text citations to match accordingly. Please see our Supporting Information guidelines for more information: http://journals.plos.org/plosone/s/supporting-information.

Answer: Captions for the supporting information is now provided at the end of the manuscript.

- 5. PLOS ONE now requires that authors provide the original uncropped and unadjusted images underlying all blot or gel results reported in a submission’s figures or Supporting Information files. This policy and the journal’s other requirements for blot/gel reporting and figure preparation are described in detail at https://journals.plos.org/plosone/s/figures#loc-blot-and-gel-reporting-requirements and https://journals.plos.org/plosone/s/figures#loc-preparing-figures-from-image-files. When you submit your revised manuscript, please ensure that your figures adhere fully to these guidelines and provide the original underlying images for all blot or gel data reported in your submission. See the following link for instructions on providing the original image data: [https://journals.plos.org/plosone/s/figures#loc-original-images-for-blots-and-gels](https://journals.plos.org/plosone/s/figures%252523loc-original-images-for-blots-and-gels).

Answer: The original uncropped and unadjusted image of the gel (related to Figure 1 in the manuscript) is now provided as supplementary Figure S6 in the Supplementary information file.

- In your cover letter, please note whether your blot/gel image data are in Supporting Information or posted at a public data repository, provide the repository URL if relevant, and provide specific details as to which raw blot/gel images, if any, are not available. Email us at plosone@plos.org if you have any questions.

Answer: The blot/gel image data is provided in the Supporting Information.

5. Review Comments to the Author

Please use the space provided to explain your answers to the questions above. You may also include additional comments for the author, including concerns about dual publication, research ethics, or publication ethics. (Please upload your review as an attachment if it exceeds 20,000 characters)

Reviewer #1: Comments:

It is interesting to reveal the effect of unpleasant smell such as TEMED on the structure and function of proteins. The work by Seraj et al. describes TEMED could inhibit the fibril formation of Hen egg white lysozyme (HEWL). Further, they identified the binding site(s) of TEMED and HEWL, and the hotspot(s) required to inhibit fibril formation. In addition, the binding of TEMED and HEWL increased the activity and thermal stability of HEWL. In conclusion, the role of the "smell of death" could serve as an important signal molecule to affect the activity and stability of HEWL. I think the work may be interesting for the potential readers of the journal. However, the following issues should be clearly addressed before it is accepted for publication.

- 1. The researchers identified the binding site(s) of TEMED and HEWL, and the hotspot(s) required to inhibit fibril formation through the Ligplot analysis. Could you please kindly provide more methods/techniques such as site-directed mutagenesis to confirm your findings?

Answer: Thank you for your suggestion. However, in this study, we used X-ray crystallography with high resolution crystal structures to identify the binding site(s) and in particular the hotspot for inhibition of fibril formation in HEWL. The Ligplot images are a means to present the data obtained from our crystal structures showing residues in proximity to TEMED, their distances and types of interactions.

Additionally, as mentioned in our manuscript, the binding of TEMED increased the activity of HEWL further emphasising the position and residues involved in binding:

Additionally, according to the results obtained in this study, position A could be strongly suggested as the "hotspot" for inhibition of fibril formation in HEWL. This position is near the active site of HEWL and binding of TEMED to this position could explain why treatment of HEWL with TEMED resulted in an increased enzymatic activity. Harada *et al.* 2007, claimed that existence of lysyl residues with a positive charge at the C-terminal of α-helix A, α-helix B and α-helix C (consisting of residues 5-15, 26-36 and 89-100, respectively) contributed to the stability of helices, resulting in conformational stability of HEWL ADDIN EN.CITE <EndNote><Cite><Author>Harada, Akihito; Yagi, Hiroshi; Saito, Akira; Azakami, Hiroyuki; Kato, Akio</Author><Year>2007</Year><DisplayText>[1]</DisplayText><record><database name="11.enl" path="/Volumes/Seagate Backup Plus Drive/thesis/My EndNote Library ظشاقش .Data/11.enl">11.enl</database><source-app name="EndNote" version="19.2">EndNote</source-app><rec-number>4</rec-number><foreign-keys><key app="EN" db-id="r0xvefzp8tx9zzewepypx027dxp2wd592szw">4</key></foreign-keys><ref-type name="Journal Article">17</ref-type><contributors><authors><author><style face="normal" font="default" size="100%">Harada, Akihito</style></author><author><style face="normal" font="default" size="100%">Yagi, Hiroshi</style></author><author><style face="normal" font="default" size="100%">Saito, Akira</style></author><author><style face="normal" font="default" size="100%">Azakami, Hiroyuki</style></author><author><style face="normal" font="default" size="100%">Kato, Akio</style></author></authors></contributors><titles><title><style face="normal" font="default" size="100%">Relationship between the stability of Hen Egg-White lysozymes mutated at sites designed to interact with α-helix dipoles and their secretion amounts in yeast</style></title><secondary-title><style face="normal" font="default" size="100%">Bioscience, biotechnology, and biochemistry</style></secondary-title></titles><periodical><full-title><style face="normal" font="default" size="100%">Bioscience, biotechnology, and biochemistry</style></full-title></periodical><pages><style face="normal" font="default" size="100%">2952-2961</style></pages><volume><style face="normal" font="default" size="100%">71</style></volume><number><style face="normal" font="default" size="100%">12</style></number><dates><year><style face="normal" font="default" size="100%">2007</style></year></dates><isbn><style face="normal" font="default" size="100%">0916-8451</style></isbn><urls/></record></Cite></EndNote>[1]. Kawamura *et al*. 2008, stated that Arg114 and Phe34 are two residues which are responsible for the productive binding at right-sided binding site of HEWL and that the CH-π interaction between the guanidyl side chain (C(NH_2_)_2_) of Arg114 and the phenyl ring of Phe34 on the molecular surface could stabilize the Arg114 side chain and fix it to accept the substrate molecule via the right-sided mode ADDIN EN.CITE <EndNote><Cite><Author>Kawamura, Shunsuke; Chijiiwa, Yuki; Minematsu, Toshie; Fukamizo, Tamo; VÅRUM, Kjell M; Torikata, Takao</Author><Year>2008</Year><DisplayText>[2]</DisplayText><record><database name="My EndNote Librarynnnn.enl" path="/Users/zahraseraj/Documents/My EndNote Librarynnnn.enl">My EndNote Librarynnnn.enl</database><source-app name="EndNote" version="19.2">EndNote</source-app><rec-number>9</rec-number><foreign-keys><key app="EN" db-id="9fsz20stms52ztevr2jpws54v5da9xzp205s">9</key></foreign-keys><ref-type name="Journal Article">17</ref-type><contributors><authors><author><style face="normal" font="default" size="100%">Kawamura, Shunsuke</style></author><author><style face="normal" font="default" size="100%">Chijiiwa, Yuki</style></author><author><style face="normal" font="default" size="100%">Minematsu, Toshie</style></author><author><style face="normal" font="default" size="100%">Fukamizo, Tamo</style></author><author><style face="normal" font="default" size="100%">VÅRUM, Kjell M</style></author><author><style face="normal" font="default" size="100%">Torikata, Takao</style></author></authors></contributors><titles><title><style face="normal" font="default" size="100%">The role of Arg114 at subsites E and F in reactions catalyzed by hen egg-white lysozyme</style></title><secondary-title><style face="normal" font="default" size="100%">Bioscience, biotechnology, and biochemistry</style></secondary-title></titles><periodical><full-title><style face="normal" font="default" size="100%">Bioscience, biotechnology, and biochemistry</style></full-title></periodical><pages><style face="normal" font="default" size="100%">823-832</style></pages><volume><style face="normal" font="default" size="100%">72</style></volume><number><style face="normal" font="default" size="100%">3</style></number><dates><year><style face="normal" font="default" size="100%">2008</style></year></dates><isbn><style face="normal" font="default" size="100%">0916-8451</style></isbn><urls/></record></Cite></EndNote>[2]. Accordingly, the interaction between Arg114 and Phe34 is suggested to be the important structural factor in maintaining the native conformation of HEWL. Based on their results, Arg114 in HEWL is thought to play an important role in lysozyme catalysis, especially in transglycosylation**.** Now looking at our results of the enzymatic activity test and the structure of HEWL treated with TEMED, the presence of TEMED near Phe34 (with hydrophobic interactions) could have an effect on the stabilization of Arg114. Additionally, it is plausible to say that the CH-π interaction between the methylamine (N(CH₃)₂) groups in TEMED (of which there are two) and phenyl ring in Phe34 (providing the π system), in addition to the CH-π interaction between the guanidyl side chain of Arg114 and again that of the phenyl ring of Phe34, are the main contributors in enhancing the ability of HEWL to accept its substrate, such that we see greater enzymatic activity in HEWL in the presence of TEMED and greater intrinsic fluorescence emission.

- 2. Is it specific for TEMED to inhibit fibril formation and increase the activity and thermal stability of HEWL? Does it affect the structure and activity of other proteins such as BSA?

Answer: Since the aim of this research was to use HEWL as a model protein for fibrillation studies, we were not concerned with the effect of TEMED on other proteins, but rather the effect of different small molecules on inhibition of HEWL fibril formation. However, we have preliminary DSF results showing that the presence of different concentrations of TEMED for two different purified proteins, Thiosulfate transferase (hTst) and 1-deoxy-D-xylulose 5-phosphate synthase from *Mycobacterium tuberculosis* (mtDXS), either showed no thermal stability changes or resulted in decreased Tm, respectively (please see Figure below).

Therefore, it appears so far that TEMED is specific for increasing the thermal stability of HEWL.

The DSF results are provided to answer the respectable reviewer’s question, but is preliminary and preferably not to be included in this manuscript.

Additionally, Anthony *et al*., reported from their study that in contrast to the effects of TEMED, which we observed on HEWL, all the three similar polyamines listed in their study including putrescine, spermidine and spermine resulted in an increased alpha synuclein aggregation ADDIN EN.CITE <EndNote><Cite><Author>Antony, T.; Hoyer, W.; Cherny, D.; Heim, G.; Jovin, T. M.; Subramaniam, V.</Author><Year>2003</Year><RecNum>19</RecNum><DisplayText>[3]</DisplayText><record><database name="My EndNote Library.enl" path="/Users/zahraseraj/Documents/My EndNote Library.enl">My EndNote Library.enl</database><source-app name="EndNote" version="19.2">EndNote</source-app><rec-number>19</rec-number><foreign-keys><key app="EN" db-id="0er29ptt6d9seaerfprxfrd02wsrs0pvzwfr">19</key></foreign-keys><ref-type name="Journal Article">17</ref-type><contributors><authors><author><style face="normal" font="default" size="100%">Antony, T.</style></author><author><style face="normal" font="default" size="100%">Hoyer, W.</style></author><author><style face="normal" font="default" size="100%">Cherny, D.</style></author><author><style face="normal" font="default" size="100%">Heim, G.</style></author><author><style face="normal" font="default" size="100%">Jovin, T. M.</style></author><author><style face="normal" font="default" size="100%">Subramaniam, V.</style></author></authors></contributors><auth-address><style face="normal" font="default" size="100%">Department of Molecular Biology, Max Planck Institute for Biophysical Chemistry, Am Fassberg 11, Goettingen D-37077, Germany.</style></auth-address><titles><title><style face="normal" font="default" size="100%">Cellular polyamines promote the aggregation of alpha-synuclein</style></title><secondary-title><style face="normal" font="default" size="100%">J Biol Chem</style></secondary-title><alt-title><style face="normal" font="default" size="100%">The Journal of biological chemistry</style></alt-title></titles><periodical><full-title><style face="normal" font="default" size="100%">J Biol Chem</style></full-title><abbr-1><style face="normal" font="default" size="100%">The Journal of biological chemistry</style></abbr-1></periodical><alt-periodical><full-title><style face="normal" font="default" size="100%">J Biol Chem</style></full-title><abbr-1><style face="normal" font="default" size="100%">The Journal of biological chemistry</style></abbr-1></alt-periodical><pages><style face="normal" font="default" size="100%">3235-40</style></pages><volume><style face="normal" font="default" size="100%">278</style></volume><number><style face="normal" font="default" size="100%">5</style></number><edition><style face="normal" font="default" size="100%">2002/11/19</style></edition><keywords><keyword><style face="normal" font="default" size="100%">Binding Sites</style></keyword><keyword><style face="normal" font="default" size="100%">Circular Dichroism</style></keyword><keyword><style face="normal" font="default" size="100%">Cloning, Molecular</style></keyword><keyword><style face="normal" font="default" size="100%">Escherichia coli/genetics</style></keyword><keyword><style face="normal" font="default" size="100%">Humans</style></keyword><keyword><style face="normal" font="default" size="100%">Microscopy, Atomic Force</style></keyword><keyword><style face="normal" font="default" size="100%">Microscopy, Electron</style></keyword><keyword><style face="normal" font="default" size="100%">Nerve Tissue Proteins/*chemistry/*ultrastructure</style></keyword><keyword><style face="normal" font="default" size="100%">Parkinson Disease</style></keyword><keyword><style face="normal" font="default" size="100%">Phosphoproteins/chemistry</style></keyword><keyword><style face="normal" font="default" size="100%">Protein Conformation</style></keyword><keyword><style face="normal" font="default" size="100%">Putrescine/*physiology</style></keyword><keyword><style face="normal" font="default" size="100%">Recombinant Proteins/chemistry</style></keyword><keyword><style face="normal" font="default" size="100%">Spectrometry, Fluorescence</style></keyword><keyword><style face="normal" font="default" size="100%">Spermidine/*physiology</style></keyword><keyword><style face="normal" font="default" size="100%">Spermine/*physiology</style></keyword><keyword><style face="normal" font="default" size="100%">Synucleins</style></keyword><keyword><style face="normal" font="default" size="100%">alpha-Synuclein</style></keyword></keywords><dates><year><style face="normal" font="default" size="100%">2003</style></year><pub-dates><date><style face="normal" font="default" size="100%">Jan 31</style></date></pub-dates></dates><isbn><style face="normal" font="default" size="100%">0021-9258 (Print)0021-9258</style></isbn><accession-num><style face="normal" font="default" size="100%">12435752</style></accession-num><abstract><style face="normal" font="default" size="100%">The cellular polyamines putrescine, spermidine, and spermine accelerate the aggregation and fibrillization of alpha-synuclein, the major protein component of Lewy bodies associated with Parkinson's disease. Circular dichroism and fluorometric thioflavin T kinetic studies showed a transition of alpha-synuclein from unaggregated to highly aggregated states, characterized by lag and transition phases. In the presence of polyamines, both the lag and transition times were significantly shorter. All three polyamines accelerated the aggregation and fibrillization of alpha-synuclein to a degree that increased with the total charge, length, and concentration of the polyamine. Electron and scanning force microscopy of the reaction products after the lag phase revealed the presence of aggregated particles (protofibrils) and small fibrils. At the end of the transition phase, alpha-synuclein formed long fibrils in all cases, although some morphological variations were apparent. In the presence of polyamines, fibrils formed large networks leading ultimately to condensed aggregates. In the absence of polyamines, fibrils were mostly isolated. We conclude that the polyamines at physiological concentrations can modulate the propensity of alpha-synuclein to form fibrils and may hence play a role in the formation of cytosolic alpha-synuclein aggregates.</style></abstract><notes><style face="normal" font="default" size="100%">Antony, ThomasHoyer, WolfgangCherny, DmitryHeim, GudrunJovin, Thomas MSubramaniam, VinodJournal ArticleResearch Support, Non-U.S. Gov'tUnited StatesJ Biol Chem. 2003 Jan 31;278(5):3235-40. doi: 10.1074/jbc.M208249200. Epub 2002 Nov 14.</style></notes><urls/><electronic-resource-num><style face="normal" font="default" size="100%">10.1074/jbc.M208249200</style></electronic-resource-num><remote-database-provider><style face="normal" font="default" size="100%">NLM</style></remote-database-provider><language><style face="normal" font="default" size="100%">eng</style></language></record></Cite></EndNote>[3]. Another report from Luo *et al*. showed that cellular polyamines promote amyloid-Beta (Aβ) peptide fibrillation ADDIN EN.CITE <EndNote><Cite><Author>Luo, J.; Yu, C. H.; Yu, H.; Borstnar, R.; Kamerlin, S. C.; Graslund, A.; Abrahams, J. P.; Warmlander, S. K.</Author><Year>2013</Year><RecNum>30</RecNum><DisplayText>[4]</DisplayText><record><database name="My EndNote Library.enl" path="/Users/zahraseraj/Documents/My EndNote Library.enl">My EndNote Library.enl</database><source-app name="EndNote" version="19.2">EndNote</source-app><rec-number>30</rec-number><foreign-keys><key app="EN" db-id="0er29ptt6d9seaerfprxfrd02wsrs0pvzwfr">30</key></foreign-keys><ref-type name="Journal Article">17</ref-type><contributors><authors><author><style face="normal" font="default" size="100%">Luo, J.</style></author><author><style face="normal" font="default" size="100%">Yu, C. H.</style></author><author><style face="normal" font="default" size="100%">Yu, H.</style></author><author><style face="normal" font="default" size="100%">Borstnar, R.</style></author><author><style face="normal" font="default" size="100%">Kamerlin, S. C.</style></author><author><style face="normal" font="default" size="100%">Graslund, A.</style></author><author><style face="normal" font="default" size="100%">Abrahams, J. P.</style></author><author><style face="normal" font="default" size="100%">Warmlander, S. K.</style></author></authors></contributors><auth-address><style face="normal" font="default" size="100%">Gorlaeus Laboratory, Leiden Institute of Chemistry, Leiden University, 2300RA Leiden, The Netherlands.</style></auth-address><titles><title><style face="normal" font="default" size="100%">Cellular polyamines promote amyloid-beta (Abeta) peptide fibrillation and modulate the aggregation pathways</style></title><secondary-title><style face="normal" font="default" size="100%">ACS Chem Neurosci</style></secondary-title><alt-title><style face="normal" font="default" size="100%">ACS chemical neuroscience</style></alt-title></titles><periodical><full-title><style face="normal" font="default" size="100%">ACS Chem Neurosci</style></full-title><abbr-1><style face="normal" font="default" size="100%">ACS chemical neuroscience</style></abbr-1></periodical><alt-periodical><full-title><style face="normal" font="default" size="100%">ACS Chem Neurosci</style></full-title><abbr-1><style face="normal" font="default" size="100%">ACS chemical neuroscience</style></abbr-1></alt-periodical><pages><style face="normal" font="default" size="100%">454-62</style></pages><volume><style face="normal" font="default" size="100%">4</style></volume><number><style face="normal" font="default" size="100%">3</style></number><edition><style face="normal" font="default" size="100%">2013/03/21</style></edition><keywords><keyword><style face="normal" font="default" size="100%">Alzheimer Disease/metabolism</style></keyword><keyword><style face="normal" font="default" size="100%">Amyloid/*metabolism</style></keyword><keyword><style face="normal" font="default" size="100%">Amyloid beta-Peptides/chemistry/*metabolism</style></keyword><keyword><style face="normal" font="default" size="100%">Humans</style></keyword><keyword><style face="normal" font="default" size="100%">Peptide Fragments/chemistry/*metabolism</style></keyword><keyword><style face="normal" font="default" size="100%">Polyamines/chemistry/metabolism</style></keyword><keyword><style face="normal" font="default" size="100%">Putrescine/chemistry/*metabolism</style></keyword><keyword><style face="normal" font="default" size="100%">Signal Transduction/*physiology</style></keyword><keyword><style face="normal" font="default" size="100%">Spermidine/chemistry/*metabolism</style></keyword><keyword><style face="normal" font="default" size="100%">Spermine/chemistry/*metabolism</style></keyword></keywords><dates><year><style face="normal" font="default" size="100%">2013</style></year><pub-dates><date><style face="normal" font="default" size="100%">Mar 20</style></date></pub-dates></dates><isbn><style face="normal" font="default" size="100%">1948-7193</style></isbn><accession-num><style face="normal" font="default" size="100%">23509981</style></accession-num><abstract><style face="normal" font="default" size="100%">The cellular polyamines spermine, spermidine, and their metabolic precursor putrescine, have long been associated with cell-growth, tumor-related gene regulations, and Alzheimer's disease. Here, we show by in vitro spectroscopy and AFM imaging, that these molecules promote aggregation of amyloid-beta (Abeta) peptides into fibrils and modulate the aggregation pathways. NMR measurements showed that the three polyamines share a similar binding mode to monomeric Abeta(1-40) peptide. Kinetic ThT studies showed that already very low polyamine concentrations promote amyloid formation: addition of 10 muM spermine (normal intracellular concentration is ~1 mM) significantly decreased the lag and transition times of the aggregation process. Spermidine and putrescine additions yielded similar but weaker effects. CD measurements demonstrated that the three polyamines induce different aggregation pathways, involving different forms of induced secondary structure. This is supported by AFM images showing that the three polyamines induce Abeta(1-40) aggregates with different morphologies. The results reinforce the notion that designing suitable ligands which modulate the aggregation of Abeta peptides toward minimally toxic pathways may be a possible therapeutic strategy for Alzheimer's disease.</style></abstract><notes><style face="normal" font="default" size="100%">1948-7193Luo, JinghuiYu, Chien-HungYu, HuixinBorstnar, RokKamerlin, Shina C LGraslund, AstridAbrahams, Jan PieterWarmlander, Sebastian K T SJournal ArticleResearch Support, Non-U.S. Gov'tUnited StatesACS Chem Neurosci. 2013 Mar 20;4(3):454-62. doi: 10.1021/cn300170x. Epub 2013 Jan 16.</style></notes><urls/><custom2><style face="normal" font="default" size="100%">PMC3605822</style></custom2><electronic-resource-num><style face="normal" font="default" size="100%">10.1021/cn300170x</style></electronic-resource-num><remote-database-provider><style face="normal" font="default" size="100%">NLM</style></remote-database-provider><language><style face="normal" font="default" size="100%">eng</style></language></record></Cite></EndNote>[4]. Furthermore, it is reported that the acceleration of aggregation by certain polyamines suggest that they may also facilitate neurodegeneration ADDIN EN.CITE <EndNote><Cite><Author>Antony, T.; Hoyer, W.; Cherny, D.; Heim, G.; Jovin, T. M.; Subramaniam, V.</Author><Year>2003</Year><RecNum>19</RecNum><DisplayText>[3]</DisplayText><record><database name="My EndNote Library.enl" path="/Users/zahraseraj/Documents/My EndNote Library.enl">My EndNote Library.enl</database><source-app name="EndNote" version="19.2">EndNote</source-app><rec-number>19</rec-number><foreign-keys><key app="EN" db-id="0er29ptt6d9seaerfprxfrd02wsrs0pvzwfr">19</key></foreign-keys><ref-type name="Journal Article">17</ref-type><contributors><authors><author><style face="normal" font="default" size="100%">Antony, T.</style></author><author><style face="normal" font="default" size="100%">Hoyer, W.</style></author><author><style face="normal" font="default" size="100%">Cherny, D.</style></author><author><style face="normal" font="default" size="100%">Heim, G.</style></author><author><style face="normal" font="default" size="100%">Jovin, T. M.</style></author><author><style face="normal" font="default" size="100%">Subramaniam, V.</style></author></authors></contributors><auth-address><style face="normal" font="default" size="100%">Department of Molecular Biology, Max Planck Institute for Biophysical Chemistry, Am Fassberg 11, Goettingen D-37077, Germany.</style></auth-address><titles><title><style face="normal" font="default" size="100%">Cellular polyamines promote the aggregation of alpha-synuclein</style></title><secondary-title><style face="normal" font="default" size="100%">J Biol Chem</style></secondary-title><alt-title><style face="normal" font="default" size="100%">The Journal of biological chemistry</style></alt-title></titles><periodical><full-title><style face="normal" font="default" size="100%">J Biol Chem</style></full-title><abbr-1><style face="normal" font="default" size="100%">The Journal of biological chemistry</style></abbr-1></periodical><alt-periodical><full-title><style face="normal" font="default" size="100%">J Biol Chem</style></full-title><abbr-1><style face="normal" font="default" size="100%">The Journal of biological chemistry</style></abbr-1></alt-periodical><pages><style face="normal" font="default" size="100%">3235-40</style></pages><volume><style face="normal" font="default" size="100%">278</style></volume><number><style face="normal" font="default" size="100%">5</style></number><edition><style face="normal" font="default" size="100%">2002/11/19</style></edition><keywords><keyword><style face="normal" font="default" size="100%">Binding Sites</style></keyword><keyword><style face="normal" font="default" size="100%">Circular Dichroism</style></keyword><keyword><style face="normal" font="default" size="100%">Cloning, Molecular</style></keyword><keyword><style face="normal" font="default" size="100%">Escherichia coli/genetics</style></keyword><keyword><style face="normal" font="default" size="100%">Humans</style></keyword><keyword><style face="normal" font="default" size="100%">Microscopy, Atomic Force</style></keyword><keyword><style face="normal" font="default" size="100%">Microscopy, Electron</style></keyword><keyword><style face="normal" font="default" size="100%">Nerve Tissue Proteins/*chemistry/*ultrastructure</style></keyword><keyword><style face="normal" font="default" size="100%">Parkinson Disease</style></keyword><keyword><style face="normal" font="default" size="100%">Phosphoproteins/chemistry</style></keyword><keyword><style face="normal" font="default" size="100%">Protein Conformation</style></keyword><keyword><style face="normal" font="default" size="100%">Putrescine/*physiology</style></keyword><keyword><style face="normal" font="default" size="100%">Recombinant Proteins/chemistry</style></keyword><keyword><style face="normal" font="default" size="100%">Spectrometry, Fluorescence</style></keyword><keyword><style face="normal" font="default" size="100%">Spermidine/*physiology</style></keyword><keyword><style face="normal" font="default" size="100%">Spermine/*physiology</style></keyword><keyword><style face="normal" font="default" size="100%">Synucleins</style></keyword><keyword><style face="normal" font="default" size="100%">alpha-Synuclein</style></keyword></keywords><dates><year><style face="normal" font="default" size="100%">2003</style></year><pub-dates><date><style face="normal" font="default" size="100%">Jan 31</style></date></pub-dates></dates><isbn><style face="normal" font="default" size="100%">0021-9258 (Print) 0021-9258</style></isbn><accession-num><style face="normal" font="default" size="100%">12435752</style></accession-num><abstract><style face="normal" font="default" size="100%">The cellular polyamines putrescine, spermidine, and spermine accelerate the aggregation and fibrillization of alpha-synuclein, the major protein component of Lewy bodies associated with Parkinson's disease. Circular dichroism and fluorometric thioflavin T kinetic studies showed a transition of alpha-synuclein from unaggregated to highly aggregated states, characterized by lag and transition phases. In the presence of polyamines, both the lag and transition times were significantly shorter. All three polyamines accelerated the aggregation and fibrillization of alpha-synuclein to a degree that increased with the total charge, length, and concentration of the polyamine. Electron and scanning force microscopy of the reaction products after the lag phase revealed the presence of aggregated particles (protofibrils) and small fibrils. At the end of the transition phase, alpha-synuclein formed long fibrils in all cases, although some morphological variations were apparent. In the presence of polyamines, fibrils formed large networks leading ultimately to condensed aggregates. In the absence of polyamines, fibrils were mostly isolated. We conclude that the polyamines at physiological concentrations can modulate the propensity of alpha-synuclein to form fibrils and may hence play a role in the formation of cytosolic alpha-synuclein aggregates.</style></abstract><notes><style face="normal" font="default" size="100%">Antony, Thomas Hoyer, Wolfgang Cherny, Dmitry Heim, Gudrun Jovin, Thomas M Subramaniam, Vinod Journal Article Research Support, Non-U.S. Gov't United States J Biol Chem. 2003 Jan 31;278(5):3235-40. doi: 10.1074/jbc.M208249200. Epub 2002 Nov 14.</style></notes><urls/><electronic-resource-num><style face="normal" font="default" size="100%">10.1074/jbc.M208249200</style></electronic-resource-num><remote-database-provider><style face="normal" font="default" size="100%">NLM</style></remote-database-provider><language><style face="normal" font="default" size="100%">eng</style></language></record></Cite></EndNote>[3].

**DSF results of 1-deoxy-D-xylulose 5-phosphate synthase from *Mycobacterium tuberculosis* (mtDXS) and Thiosulfate transferase (hTst) in either the absence or presence of different concentrations of TEMED in solution.** (a) 1-deoxy-D-xylulose 5-phosphate synthase from *Mycobacterium tuberculosis* (mtDXS). (b) Thiosulfate transferase (hTst).

- 3. Is there other diamine affecting the activity and structure of HEWL?

Answer: Yes, there is a report from Okanojo, *et al*., at 2005 which showed that diamines prevent thermal aggregation and inactivation of lysozyme ADDIN EN.CITE <EndNote><Cite><Author>Okanojo, M.; Shiraki, K.; Kudou, M.; Nishikori, S.; Takagi, M.</Author><Year>2005</Year><RecNum>20</RecNum><DisplayText>[5]</DisplayText><record><database name="My EndNote Library.enl" path="/Users/zahraseraj/Documents/My EndNote Library.enl">My EndNote Library.enl</database><source-app name="EndNote" version="19.2">EndNote</source-app><rec-number>20</rec-number><foreign-keys><key app="EN" db-id="0er29ptt6d9seaerfprxfrd02wsrs0pvzwfr">20</key></foreign-keys><ref-type name="Journal Article">17</ref-type><contributors><authors><author><style face="normal" font="default" size="100%">Okanojo, M.</style></author><author><style face="normal" font="default" size="100%">Shiraki, K.</style></author><author><style face="normal" font="default" size="100%">Kudou, M.</style></author><author><style face="normal" font="default" size="100%">Nishikori, S.</style></author><author><style face="normal" font="default" size="100%">Takagi, M.</style></author></authors></contributors><auth-address><style face="normal" font="default" size="100%">School of Materials Science, Japan Advanced Institute of Science and Technology, 1-1 Asahidai, Nomi, Ishikawa 923-1292, Japan.</style></auth-address><titles><title><style face="normal" font="default" size="100%">Diamines prevent thermal aggregation and inactivation of lysozyme</style></title><secondary-title><style face="normal" font="default" size="100%">J Biosci Bioeng</style></secondary-title><alt-title><style face="normal" font="default" size="100%">Journal of bioscience and bioengineering</style></alt-title></titles><periodical><full-title><style face="normal" font="default" size="100%">J Biosci Bioeng</style></full-title><abbr-1><style face="normal" font="default" size="100%">Journal of bioscience and bioengineering</style></abbr-1></periodical><alt-periodical><full-title><style face="normal" font="default" size="100%">J Biosci Bioeng</style></full-title><abbr-1><style face="normal" font="default" size="100%">Journal of bioscience and bioengineering</style></abbr-1></alt-periodical><pages><style face="normal" font="default" size="100%">556-61</style></pages><volume><style face="normal" font="default" size="100%">100</style></volume><number><style face="normal" font="default" size="100%">5</style></number><edition><style face="normal" font="default" size="100%">2005/12/31</style></edition><keywords><keyword><style face="normal" font="default" size="100%">Amines/*chemistry</style></keyword><keyword><style face="normal" font="default" size="100%">Animals</style></keyword><keyword><style face="normal" font="default" size="100%">Chickens</style></keyword><keyword><style face="normal" font="default" size="100%">Enzyme Activation</style></keyword><keyword><style face="normal" font="default" size="100%">Molecular Chaperones/*chemistry</style></keyword><keyword><style face="normal" font="default" size="100%">Muramidase/*chemistry</style></keyword><keyword><style face="normal" font="default" size="100%">*Protein Folding</style></keyword></keywords><dates><year><style face="normal" font="default" size="100%">2005</style></year><pub-dates><date><style face="normal" font="default" size="100%">Nov</style></date></pub-dates></dates><isbn><style face="normal" font="default" size="100%">1389-1723 (Print)1347-4421</style></isbn><accession-num><style face="normal" font="default" size="100%">16384796</style></accession-num><abstract><style face="normal" font="default" size="100%">Protein aggregation is a major obstacle in both biological applications and biomedical fields involving proteins. In this study, we investigated the essential structure of small additives that function as chemical chaperones. Aggregation-suppressing competent additives were 1,3-diaminopropane, 1,4-diaminobutane, and 1,5-diaminopentane, which suppressed aggregation in the given order; whereas no diols or monoamines prevented the thermal aggregation and the inactivation of lysozyme. The heat-inactivation rate of lysozyme with 1,3-diaminopropane was almost identical to that of lysozyme with spermine and arginine ethylester, which are the most prominent additives reported yet.</style></abstract><notes><style face="normal" font="default" size="100%">Okanojo, MasahiroShiraki, KentaroKudou, MotonoriNishikori, ShingoTakagi, MasahiroJournal ArticleResearch Support, Non-U.S. Gov'tJapanJ Biosci Bioeng. 2005 Nov;100(5):556-61. doi: 10.1263/jbb.100.556.</style></notes><urls/><electronic-resource-num><style face="normal" font="default" size="100%">10.1263/jbb.100.556</style></electronic-resource-num><remote-database-provider><style face="normal" font="default" size="100%">NLM</style></remote-database-provider><language><style face="normal" font="default" size="100%">eng</style></language></record></Cite></EndNote>[5].

- 4. Could you explain why the residues Lys33, Phe34, Glu35 and Asn37 are important for the binding of TEMED and HEWL?

Answer: As we mentioned in our manuscript, Kawamura *et al*. 2008 ADDIN EN.CITE <EndNote><Cite><Author>Kawamura, S.; Chijiiwa, Y.; Minematsu, T.; Fukamizo, T.; Varum, K. M.; Torikata, T.</Author><Year>2008</Year><RecNum>27</RecNum><DisplayText>[6]</DisplayText><record><database name="My EndNote Library.enl" path="/Users/zahraseraj/Documents/My EndNote Library.enl">My EndNote Library.enl</database><source-app name="EndNote" version="19.2">EndNote</source-app><rec-number>27</rec-number><foreign-keys><key app="EN" db-id="0er29ptt6d9seaerfprxfrd02wsrs0pvzwfr">27</key></foreign-keys><ref-type name="Journal Article">17</ref-type><contributors><authors><author><style face="normal" font="default" size="100%">Kawamura, S.</style></author><author><style face="normal" font="default" size="100%">Chijiiwa, Y.</style></author><author><style face="normal" font="default" size="100%">Minematsu, T.</style></author><author><style face="normal" font="default" size="100%">Fukamizo, T.</style></author><author><style face="normal" font="default" size="100%">Varum, K. M.</style></author><author><style face="normal" font="default" size="100%">Torikata, T.</style></author></authors></contributors><auth-address><style face="normal" font="default" size="100%">Department of Bioscience, School of Agriculture, Tokai University, Aso, Kumamoto 869-1404, Japan. kawamura@agri.u-tokai.ac.jp</style></auth-address><titles><title><style face="normal" font="default" size="100%">The role of Arg114 at subsites E and F in reactions catalyzed by hen egg-white lysozyme</style></title><secondary-title><style face="normal" font="default" size="100%">Biosci Biotechnol Biochem</style></secondary-title><alt-title><style face="normal" font="default" size="100%">Bioscience, biotechnology, and biochemistry</style></alt-title></titles><periodical><full-title><style face="normal" font="default" size="100%">Biosci Biotechnol Biochem</style></full-title><abbr-1><style face="normal" font="default" size="100%">Bioscience, biotechnology, and biochemistry</style></abbr-1></periodical><alt-periodical><full-title><style face="normal" font="default" size="100%">Biosci Biotechnol Biochem</style></full-title><abbr-1><style face="normal" font="default" size="100%">Bioscience, biotechnology, and biochemistry</style></abbr-1></alt-periodical><pages><style face="normal" font="default" size="100%">823-32</style></pages><volume><style face="normal" font="default" size="100%">72</style></volume><number><style face="normal" font="default" size="100%">3</style></number><edition><style face="normal" font="default" size="100%">2008/03/08</style></edition><keywords><keyword><style face="normal" font="default" size="100%">Amino Acid Substitution</style></keyword><keyword><style face="normal" font="default" size="100%">Animals</style></keyword><keyword><style face="normal" font="default" size="100%">Arginine/*chemistry</style></keyword><keyword><style face="normal" font="default" size="100%">Catalysis</style></keyword><keyword><style face="normal" font="default" size="100%">Catalytic Domain</style></keyword><keyword><style face="normal" font="default" size="100%">Chickens</style></keyword><keyword><style face="normal" font="default" size="100%">Female</style></keyword><keyword><style face="normal" font="default" size="100%">Kinetics</style></keyword><keyword><style face="normal" font="default" size="100%">Magnetic Resonance Spectroscopy</style></keyword><keyword><style face="normal" font="default" size="100%">Muramidase/chemistry/*metabolism</style></keyword><keyword><style face="normal" font="default" size="100%">Protein Binding</style></keyword><keyword><style face="normal" font="default" size="100%">Protein Conformation</style></keyword></keywords><dates><year><style face="normal" font="default" size="100%">2008</style></year><pub-dates><date><style face="normal" font="default" size="100%">Mar</style></date></pub-dates></dates><isbn><style face="normal" font="default" size="100%">0916-8451</style></isbn><accession-num><style face="normal" font="default" size="100%">18323666</style></accession-num><abstract><style face="normal" font="default" size="100%">To understand better the role of subsites E and F in lysozyme-catalyzed reactions, mutant enzymes, in which Arg114, located on the right side of subsites E and F in hen egg-white lysozyme (HEL), was replaced with Lys, His, or Ala, were prepared. Replacement of Arg114 with His or Ala decreased hydrolytic activity toward an artificial substrate, glycol chitin, while replacement with Lys had little effect. Kinetic analysis with the substrate N-acetylglucosamine pentamer, (GlcNAc)(5), revealed that the replacement for the Arg residue reduced the binding free energies of E-F sites and the rate constant of transglycosylation. The rate constant of transglycosylation for R114A was about half of that for the wild-type enzyme. (1)H-NMR analysis of R114H and R114A indicated that the structural changes induced by the mutations were not restricted to the region surrounding Arg114, but rather extended to the aromatic side chains of Phe34 and Trp123, of which the signals are connected with each other through nuclear Overhauser effect (NOE) in the wild-type. We speculate that such a conformational change causes differences in substrate and acceptor binding at subsites E and F, lowering the efficiency of glycosyl transfer reaction of lysozyme.</style></abstract><notes><style face="normal" font="default" size="100%">1347-6947Kawamura, ShunsukeChijiiwa, YukiMinematsu, ToshieFukamizo, TamoVarum, Kjell MTorikata, TakaoJournal ArticleEnglandBiosci Biotechnol Biochem. 2008 Mar;72(3):823-32. doi: 10.1271/bbb.70694. Epub 2008 Mar 7.</style></notes><urls/><electronic-resource-num><style face="normal" font="default" size="100%">10.1271/bbb.70694</style></electronic-resource-num><remote-database-provider><style face="normal" font="default" size="100%">NLM</style></remote-database-provider><language><style face="normal" font="default" size="100%">eng</style></language></record></Cite></EndNote>[6], stated that Arg114 and Phe34 are two residues which are responsible for the productive binding at right-sided binding site of HEWL. Furthermore, the CH-Π interaction between the guanidyl side chain (C(NH_2_)_2_) of Arg114 and the phenyl ring of Phe34 on the molecular surface could stabilize the Arg114 side chain and fix it to accept the substrate molecule via the right-sided mode. Accordingly, the interaction between Arg114 and Phe34 is suggested to be the important structural factor in maintaining the proper conformation of HEWL. Based on their results, Arg114 in HEWL is thought to play an important role in lysozyme catalysis, especially in transglycosylation**.** Now looking at our results of the enzymatic activity test and the structure of HEWL treated with TEMED, the presence of TEMED near Phe34 (with hydrophobic interactions) could have an effect on the stabilization of Arg114. Additionally, it is plausible to say that the CH-Π interaction between the methylamine (N(CH₃)₂) groups in TEMED (of which there are two) and phenyl ring in Phe34 (providing the Π system) in addition to the CH-Π interaction between the guanidyl side chain of Arg114 and again that of the phenyl ring of Phe34, is the main contributor in enhancing the ability of HEWL to accept its substrate such that we see greater enzymatic activity in HEWL in the presence of TEMED and greater intrinsic fluorescence emission.

Additionally, binding of TEMED close to Glu35 as one of the residues in the active site including Glu35 and Asp52, which resulted in increasing in the activity of HEWL, was supportive data about the importance of this position.

- 5. The abstract should be revised carefully to describe the goal of this study, the main findings and the conclusion to help readers better understand the novelty of this study.

Answer: Thank you. The abstract has been improved.

- 6. The figures and tables in this manuscript could not meet the science criterion and should be thoroughly reorganized and polished to present to the readers.

Answer: Changes were applied as much as possible.

- 7. The conclusion is too long. It should be concise and highlight the most important findings and scientific significance. Please revise it carefully.

Answer: Changes were applied to the conclusion section of the manuscript.

- 8. The authors should have their work reviewed by a proper translation/reviewing service or native-English speaker before submission. The manuscript needs careful editing by someone with particular attentions to English grammar, spelling, and sentence structure so that the goal and results of the study are clear to the readers.

Answer: Thank you for your suggestion. This was done.

In my opinion, the article in the present form is not suitable for publication in PLoS One, and should undergo a round of major revision before publication at least.

Reviewer #2:

This study tried to study how "unpleasant smell" molecules (using TEMED as a model molecule) can be signal molecules and provide a structural mechanism. However, the writing of the paper is not clear even English is good. There are two major issues for this manuscript:

- 1. HEWL is more like a random model protein the authors chose to study how TEMED affects protein stability and fiber formation. There is no clear explanation why interaction of TEMED with HEWL will have a biological effect. Using model proteins to study impact of TEMED is acceptable, but making farfetched biological functions is not, and can be misleading.

Answer: As we explained in this study, TEMED was chosen because of its pungent fishlike odour and its similarity to putrescence and cadaverine as polyamines, also known as the "smell of death”, which are produced by decarboxylation of precursor amino acids by enzymes of bacterial origin. On the other hand, based on our previous study, it was interesting for us that fibril formation in HEWL, as an antibacterial enzyme, was inhibited by TEMED with properties mentioned above. Therefore, we decided to extend our study and tried to find whether or not these results were related to any potential biological effects or roles. Interesting we found, using results from a number of techniques, that TEMED was a specific ligand for HEWL.

2. Although several crystal structures were determined in complex with TEMED, the omit maps (so called Fo-Fc) and 2Fo-Fc are not provided. The authors have to provide these two maps to show that the TEMED molecules are really in the complex, instead of artifacts. For a small molecule like TEMED, arbitrarily placing atoms is not likely to impact Rfree or R in a noticeable way. By checking the B factors of some pdb structures published in this study, some TEMED molecules have high B factors, indicating low occupancy or improper model fitting.

Answer: Omit maps are now provided in the supplementary information Figures S2-S5.

**Bibliography**

ADDIN EN.REFLIST

1.	Harada, A., et al., Relationship between the stability of Hen Egg-White lysozymes mutated at sites designed to interact with α-helix dipoles and their secretion amounts in yeast. Bioscience, biotechnology, and biochemistry, 2007. **71**(12): p. 2952-2961.

2.	Kawamura, S., et al., The role of Arg114 at subsites E and F in reactions catalyzed by hen egg-white lysozyme. Bioscience, biotechnology, and biochemistry, 2008. **72**(3): p. 823-832.

3.	Antony, T., et al., Cellular polyamines promote the aggregation of alpha-synuclein. J Biol Chem, 2003. **278**(5): p. 3235-40.

4.	Luo, J., et al., Cellular polyamines promote amyloid-beta (Abeta) peptide fibrillation and modulate the aggregation pathways. ACS Chem Neurosci, 2013. **4**(3): p. 454-62.

5.	Okanojo, M., et al., Diamines prevent thermal aggregation and inactivation of lysozyme. J Biosci Bioeng, 2005. **100**(5): p. 556-61.

6.	Kawamura, S., et al., The role of Arg114 at subsites E and F in reactions catalyzed by hen egg-white lysozyme. Biosci Biotechnol Biochem, 2008. **72**(3): p. 823-32.
